# Disruptive mood dysregulation disorder, parental stress, and attachment styles

**DOI:** 10.3389/frcha.2024.1430850

**Published:** 2024-07-23

**Authors:** Marit Coldevin, Astrid Brænden, Pål Zeiner, Anne-Siri Øyen, Annika Melinder, Jan E. Stubberud

**Affiliations:** ^1^ Lovisenberg Diaconal Hospital, Nic Waals Institute, Oslo, Norway; ^2^ Department of Psychology, University of Oslo, Oslo, Norway; ^3^ Division of Mental Health and Addiction, Oslo University Hospital, Oslo, Norway

**Keywords:** DMDD, severe irritability in children, parental attachment style, parental stress, family risk factors

## Abstract

Disruptive mood dysregulation disorder (DMDD) is a relatively new diagnostic entity concerning children with chronic irritability and severe anger outbursts. Currently, there is limited knowledge about the parental factors associated with the disorder. The aim of this study was to compare stress levels and attachment styles in parents of children with DMDD with those of parents of children with other diagnoses. Our sample consisted of 218 children (6–12 years, *M*_age_ = 9.68 years) referred to child mental health outpatient clinics. Clinicians used a standardized semi-structured diagnostic interview to identify diagnoses. Parental stress levels and attachment styles were assessed using parent reports. We found that parents of children with DMDD experience significantly higher levels of parenting stress related to factors in the child than parents of children with other diagnoses. Furthermore, parents of children with DMDD show a higher association with insecure adult attachment styles than parents of children without DMDD. Finally, an adult preoccupied-ambivalent attachment style explains the variability in parental stress in the DMDD group to a large degree. We discuss how parental stress and an insecure attachment style can be associated with negative parenting practices. An implication from this study could be that treatment results might be improved by involving parents more in treatment programs for children with DMDD.

## Introduction

Disruptive mood dysregulation disorder (DMDD) was introduced as a cluster of symptoms to meet a rising clinical need to conceptualize children with severe and chronic irritability, who did not meet diagnostic criteria for other diagnoses, and to develop treatment alternatives (1). The disorder is common in clinical child populations (2–4) and is associated with high loads of psychiatric symptoms and reduced social and psychological functioning (5, 6). DMDD is considered a depressive disorder because irritability is its core symptom, but it also includes extensive behavioral symptoms, e.g., anger outbursts, that may influence the child and its surroundings to a high degree (7). There is a lack of knowledge about the parental factors that are associated with the disorder or contribute to it. Irritability and behavioral problems in children are associated with negative parenting styles (8, 9), suggesting that parental factors are relevant for understanding DMDD. Factors associated with parenting styles and DMDD need to be further explored as they may be of importance to, and inform, treatment planning for DMDD patients and their parents. Thus, in the present study, we will explore parental stress and parental attachment style in parents with children with DMDD compared with parents with children that have other mental health problems.

### Parental stress

Parental stress, or stress related to parenting tasks or adjusting to the role itself, is interesting as a parental factor as it has been found to influence the quality of caregiving (10). Specifically, high levels of perceived parental stress are associated with negative parenting styles such as less warmth and harsher discipline (11), and reduced parenting effectiveness (12). In addition, they are a source or reinforcement of behavioral problems, emotion regulation difficulties, low frustration tolerance, and reduced coping skills in the child (13). Furthermore, parental stress and child mental health problems have been found to be strongly correlated over time (10). The associations are bidirectional, reflecting their mutual influence on the interactions (13). Recent evidence also demonstrated that parents of children with neurodevelopmental disorders have higher levels of parental stress than those with healthy children, which suggests that the clinical condition of the child impacts the parentś wellbeing (14). However, parental stress load in the context of DMDD has, to our knowledge, not been widely examined. Previous research on parental stress in clinical child populations has, perhaps not surprisingly, shown that parents of children with externalizing behavior problems experience higher stress levels than parents of children with internalizing symptoms (12). As DMDD consists of internalizing (irritability/depression) and externalizing (anger outbursts) symptoms, the stress toll on these parents is most likely very high (6), potentially making effective parenting even harder for this group.

### Parental attachment styles

Another way of assessing the quality of caregiving is by using parental attachment styles, typically divided into secure and insecure styles (15–17). A basic premise of attachment theory is that internal working models of attachment remain relatively stable across the lifespan (18, 19), whereby patterns from childhood shape styles for close relationships in adulthood (15), as between a parent and a child. Insecure attachment in parents is associated with negative parental styles (20), as well as higher levels of parental stress (21). One way of categorizing insecure attachment is by sorting into four styles based on a person's model of the self (high-low self-worth) and others (high-low trust in others) (15). These types are as follows: Secure, positive model of self and others (A); Fearful, negative model of self and others (B); Preoccupied, negative model of self and positive of others (C); and Dismissing, positive model of self and negative of others (D).

Attachment patterns in parents may function as a buffer or facilitator of perceived stress as a parent (21), but for parents of children with DMDD, there is a knowledge gap regarding the relationship between these two factors. Such knowledge could help pinpoint parent management targets for this patient group. Thus, in the current study, we will (1) explore parental attachment style and parenting stress for parents with children suffering from DMDD, and compare them with parents of children with other mental health diagnoses, and (2) investigate differences in attachment styles and their potential effects on perceived parental stress in the DMDD group.

## Methods

This study was approved by the Regional Committees for Medical and Health Research Ethics (#2017/135) and is part of a registered study protocol (NCT05049356).

### Participants

Children aged 6–12 years referred for moderate-to-severe mental health problems and admitted for clinical assessment at three outpatient child and adolescent mental health clinics in Oslo, Norway, from 2019 to 2021, were invited to participate. Informed oral and written consent was obtained from the parents. Participants and their parents completed a broad clinical assessment conducted as part of each clinic's standard assessment procedure. Inclusion criteria were as follows: accepted at a mental health clinic for diagnostic evaluation, aged between 6 and 12 years, intelligence quotient (IQ) ≥ 70, and adequate Norwegian language skills for completing questionnaires and semi-structured clinical interviews. Exclusion criteria were as follows: age <6/>12 years, IQ < 69, or in active psychosis. In total, 319 children were invited to participate, of which 101 were subsequently excluded (76 declined, 18 did not meet the inclusion criteria, and 7 dropped out). The sample consisted of 218 children (60.4% boys) ranging from 6.0 to 12.9 years of age (*M* = 9.6, *SD* = 1.8), with a broad spectrum of child mental health diagnoses.

### Measures

Measures were completed as part of each child's clinical assessment.

#### Schedule for affective disorders and schizophrenia for school-aged children (K-SADS-Pl) 2016

K-SADS-PL-5 is an internationally validated semi-structured diagnostic interview corresponding with DSM-5 diagnoses and frequently applied in research and clinical practice (22). The Norwegian version of K-SADS-PL-5 was used with parents. Thirteen clinical psychologists and master’s-level psychology students administered the 2016 Norwegian version of the K-SADS-PL to parents for diagnostic evaluation. Nine percent of the interviews were independently scored by two different clinicians, demonstrating substantial agreement between the interviewers’ diagnostic evaluations overall (*к* = 0.80, 95% CI: 0.71–0.89). Interrater reliability was *к* = 0.90 (95% CI: 0.70–1.0) for DMDD, *к* = 0.78 (95% CI: 0.50–1.0) for attention deficit/hyperactivity disorders (ADHD), *к* = 0.78 (95% CI: 0.50–1.0) for oppositional defiant disorder (ODD), *к* = 0.77 (95% CI: 0.35–1.1) for depressive disorders, and *к* = 0.57 (95% CI: 0*.*13–1.0) for anxiety disorders.

#### The parenting stress index, 3rd ed.

Parenting stress index (PSI) measures parenting stress perceived by caregivers of children (0–12 years) to identify dysfunctional relationships between child and parent (23, 24). The PSI considers parental stress to be composed of two dimensions: general stress associated with parental demands and stress that is specifically derived from the child's demands. These two domains form the total scale of 101 items to which the parents reply on a Likert scale ranging from 1 (not agree at all) to 5 (totally agree). In addition, there is a life stress scale of 19 items, which provides information about parental stress caused by factors outside the relationship with the child over the past 12 months. The child domain assesses child characteristics that may contribute to overall stress and consists of six subscales (Distractibility/Hyperactivity, Adaptability, Reinforces Parent, Demandingness, Mood, and Acceptability), whereas seven subscales (Competence, Isolation, Attachment, Health, Role Restriction, Depression, and Spouse/Parenting Partner Relationship) measure parent characteristics. Test-retest reliability ranges from 0.55 to 0.82 for the child domain, 0.69 to 0.91 for the parent domain, and 0.65 to 0.96 for the total stress score (25). In this sample, the parents of 180 of the 218 children (*n* = 38 missing) answered the PSI; 80% of the respondents were mothers and Cronbach's alpha between subscales was *α* = 0.85.

#### Relationship questionnaire

The relationship questionnaire (RQ) is a four-item self-report questionnaire designed to measure adult attachment style, arranged in a nominal scale (A–D; yes or no) and a Likert scale ranging from 1 to 7 (1 = not at all as I am, 7 = very similar to me) (15). It has four measurable categories of attachment style—secure (A), fearful (B), preoccupied (C), and dismissing (D), each describing a prototypical attachment pattern as it applies to close relationships in adulthood and is described in four short paragraphs (see the full RQ questionnaire in the Supplementary Material). As shown by Brennan et al. (26), styles A, B, and C correspond, respectively, to Hazan and Shaver's (16, 27) Secure, Avoidant, and Anxious/Ambivalent styles. Bartholomew's measure adds the dismissing category (D). The retest reliability for RQ has been assessed as being in the range of 0.74–0.88 (28). The construct and external validity of the RQ have been evaluated as good (29, 30). The RQ was answered by 151 of 218 parents (67 = missing), of which 95% (*n* = 147) were mothers.

### Sociodemographic data

Parents completed a questionnaire about their living conditions and annual income. Poverty levels for families with children are incomes less than 60% of the median income in a specific country. In Norway, for a family with at least one child, the poverty level in 2019 was an annual income of <314,500 NOK (31).

### Statistical analyses

Analyses were carried out using IBM SPSS Statistics Version 28.0.0.0 (190) and R version 3.6.3 using the ggplot package for figures. The alpha level was set at 0.05. Effect sizes were measured using Cohen's *d =* 0.20 (small), 0.50 (moderate), and ≥0.80 (large effect) (32).

Descriptive statistics were used to summarize the data. Chi-square tests with Yates correction for continuity were used to examine associations between DMDD and sociodemographic factors. Analysis of variance (ANOVA) was used to examine group differences between diagnoses, attachment style, and continuous variables (e.g., parenting stress loads).

To explore differences in attachment style with regard to parental stress levels in the DMDD group, we examined (1) differences in secure and insecure styles on parental stress loads, and (2) which of the four attachment styles correlates and predicts outcomes in parental stress on child-related, parent-related, and total stress scores. Pearson's correlation analyses were used to explore associations between attachment style and parental stress in the DMDD group. Linear regression analyses were used to investigate the explained variance of attachment style on parental stress in the same group. In analyses of group differences between the DMDD and non-DMDD groups and parental stress, sex, parental living situation, and income level were included as co-variates.

## Results

The sample consisted of 218 children with different mental health diagnoses, of which 53 (24%) had DMDD and 165 (75.6%) had other diagnoses [ADHD (27%), anxiety disorders (22%), depressive disorders (8%), and oppositional defiant disorder (18%)]. When comparing DMDD and non-DMDD, the groups showed significant differences, with more boys, more single parenting, and less family income in the DMDD group (Table 1).

**Table 1:** Prevalence and sociodemographic characteristics of children with DMDD compared with a non-DMDD clinical group.

	Overall (*n* = 218)	DMDD (*n* = 53)	Non-DMDD (*n* = 165)	Statistical test or *χ*^2^	*p*-value	Effect size *φ*
Main diagnoses non-DMDD group, *n* (%)		53 (24)				
ADHD			59 (27)			
Anxiety disorders			49 (22)			
Depressive disorders			18 (8)			
Oppositional defiant disorder			39 (18)			
Age, M (SD)	9.6 (1.8)	9.3 (1.9)	9.7 (1.8)	*t*_(216)_ = 1.4	0.135	
IQ, M (SD)	99 (15.4)	99 (13.1)	99.7 (16.1)	*t*_(143)_ = 0.08	0.94	
Boys, *n* (%)	132 (60)	41 (77)	91 (55)	*χ*^2^_(2, 218)_ = 6.9	0.008^a^	0.19
Parental living situation, *n* (%)	(*n* = 213)	(*n* = 52)	(*n* = 161)	*χ*^2^_(1, 213)_ = 4.6	0.032^a^	0.14
Married/cohabitant	183 (86)	40 (77)	143 (89)			
Single parent	30 (14)	12 (23)	18 (11)			
Family income, *n* (%)	(*n* = 187)	(*n* = 44)	(*n* = 143)	*χ*^2^_(1, 187)_ = 4.8	0.028^a^	0.16
At or above poverty level	172 (92)	37 (84)	134 (94)			
Below poverty level	15 (8)	7 (16)	8 (6)			
Life stress, M (SD)	(*n* = 180)	(*n* = 40)	(*n* = 136)	*t*_(178)_ = −0.45	0.65	
	5.4 (5.9)	5.8 (6.8)	5.3 (5.6)			

ADHD, Attention deficit/hyperactivity disorders, including inattentive type, hyperactivity/impulsivity type, and ADHD not otherwise specified.

Anxiety disorders: social anxiety, separation anxiety, generalized anxiety disorder, panic disorder, and phobias. Depressive disorders (DMDD excluded): depressive episodes, dysthymia, and depression not otherwise specified. In 2019, the poverty level for families with children in Norway was defined as annual income <314,500 NOK.

^a^ Significance level < 0.05.

### Parenting stress and attachment style between the DMDD and non-DMDD groups

Analysis of parenting stress loads between children with DMDD and the non-DMDD group showed significant differences in the child domain; DMDD parents reported higher stress levels related to the child's behavior but no differences in the parental or total stress domains (see Table 2). Parents of children with DMDD had a significantly higher degree of insecure attachment style. However, only the difference in the degree of parents’ association with type D (Dismissing) between the DMDD vs. non-DMDD parenting group was statistically significant; parents of children with DMDD showed a higher degree of resemblance to style D. (See also the analysis of separate diagnoses in the non-DMDD group compared with DMDD in the Supplementary Material).

**Table 2:** Perceived stress related to the child, parent, and total stress load as reported by parents and parent attachment style for children with DMDD compared with a non-DMDD clinical group.

	Overall	*M* (SD)	*M* (SD)	Test statistic *t*(df), *F*, or *χ*^2^	*p*-value (two-tailed)	Effect sized, Φ, *η*^2^
		DMDD vs. non-DMDD clinical group				
Parental stress^a^	(*n* = 180)	DMDD (*n* = 44)	Non-DMDD (*n* = 136)			
Child domain	124.4 (25.3)	135.6 (22.2)	120.8 (25.3)	*F*(1, 177) = 8.88	0.002*	0.601
Parent domain	116.5 (27.1)	124.4 (27.4)	113.9 (26.7)	*F*(1, 177) = 3.54	0.061	0.380
Total stress load	239.9 (49.6)	254.4 (59.8)	235.2 (45.2)	*F*(1, 177) = 3.26	0.073	0.390
Parent attachment style	(*n* = 151)	(*n* = 38)	(*n* = 113)			
Secure, *n* (%)	96 (63.6)	19 (50)	77 (68)	*χ*^2^_(1, 151)_ = 4.8	0.044*	0.164
Insecure, *n* (%)	55 (36.4)	19 (50)	36 (32)			
Attachment types	(*n* = 156)	(*n* = 39)	(*n* = 117)			
Type A (secure)	5.1 (1.63)	5.1 (1.6)	5.1 (1.6)	*t*(154) = 0.141	0.888	0.026
Type B (fearful)	3.1 (1.90)	3.5 (2.0)	3.0 (1.86)	*t*(154)= −0.146	0.165	0.270
Type C (preoccupied)	2.8 (1.72)	2.8 (1.6)	2.8 (1.7)	*t*(154) = 0.081	0.936	0.015
Type D (dismissing)	2.5 (1.61)	3.1 (1.8)	2.4 (1.5)	*t*(154) = −2.192	0.033*	0.443

^a^ Controlled for sex, income, and living situation. Secure equals type A, insecure includes type B, C, and D. Attachment types refers to parental responses on a Likert scale (1 = not like me to 7 = very similar to me).

* *p <* 0.05.

An investigation of the secure vs. insecure attachment style on parenting stress levels in the overall clinical group showed that stress levels concerning the child (secure: *M* = 119.9, SD = 26.3, vs. insecure: *M* = 132.2, SD = 19.6; *t*(143) = 2.96, *p* = 0.002, *d* = 0.51), factors related to being a parent (secure: *M* = 108.17, SD = 25.1, vs. insecure: *M* = 129.4, SD = 23.7; *t*(143) = 5.00, *p* < 0.001, *d* = 0.86), and perceived total stress of parenting (secure: *M* = 228.8, SD = 47.1, vs. insecure: *M* = 257.0, SD = 51.6; *t*(143) = 3.35, *p* < 0.001, *d* = 0.57) were significantly higher for the insecure group, with moderate-to-strong effect sizes.

### Parental stress and attachment style in the DMDD group

Analysis of secure vs. insecure attachment styles for parents of children with DMDD showed that perceived stress levels related to parenting management and total stress were significantly higher, with large effect sizes for the insecure group. There were no significant differences between secure and insecure styles and stress related to factors in the child (see Table 3). When exploring the dimensional aspects of the insecure attachment style in the DMDD group more closely, style C (preoccupied) significantly correlated with stress levels in the child and parental domains, and total stress loads (see Table 4).

**Table 3:** Secure vs. insecure attachment style and parenting stress levels for parents of children with DMDD.

	Secure vs. (*n* = 18)	Insecure (*n* = 18)	*t*(34)	*p*	*d*
	M (SD)	M (SD)			
Child domain	129.2 (24.2)	138.9 (20.9)	1.28	0.207	0.429
Parent domain	112 (16.9)	137.4 (28.8)	3.22	**0**.003*	1.070
Total stress	241.2 (38.4)	278.2 (45.0)	2.62	**0**.013*	0.886

* *p* < 0.05 (two-tailed).

Bold values indicate statistical significance *p* < .05.

**Table 4:** Correlations between parental stress and attachment in the DMDD group.

	1	2	3	4	5	6	7
1. Child domain	—						
2. Parent domain	0.651*	—					
3. Total stress	0.892*	0.929*	—				
4. Type A (secure)	−0.248	−0.291	−0.301	—			
5. Type B (fearful)	−0.237	0.199	0.159	−0.237	—		
6. Type C (preoccupied)	0.351**	0.657*	0.583*	−0.311	0.243	—	
7. Type D (dismissing)	−0.217	−0.035	−0.139	−0.166	0.119	−0.038	—

* *p* < 0.01; **p < 0.05 (two-tailed).

**Figure 1:**
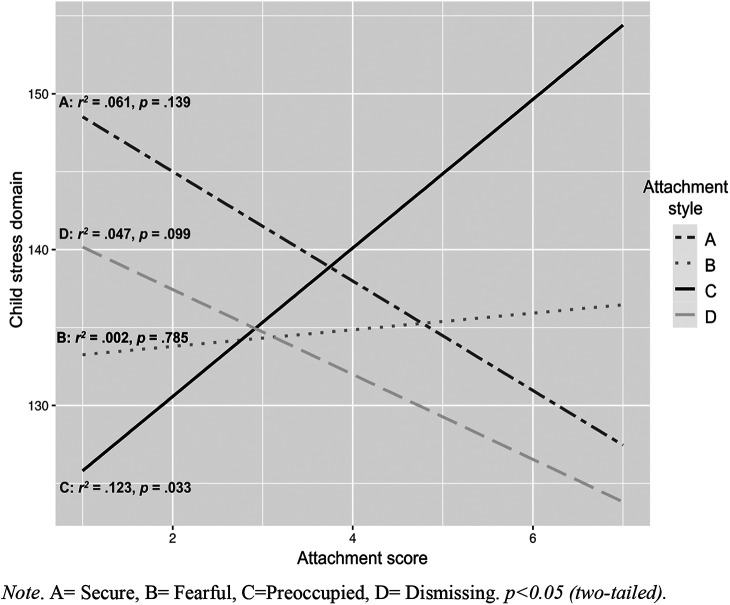
Correlates and regression coefficients of parental attachment styles and child-related stress. A, Secure; B, Fearful; C, Preoccupied; D, Dismissing. *p* < 0.05 (two-tailed).

**Figure 2:**
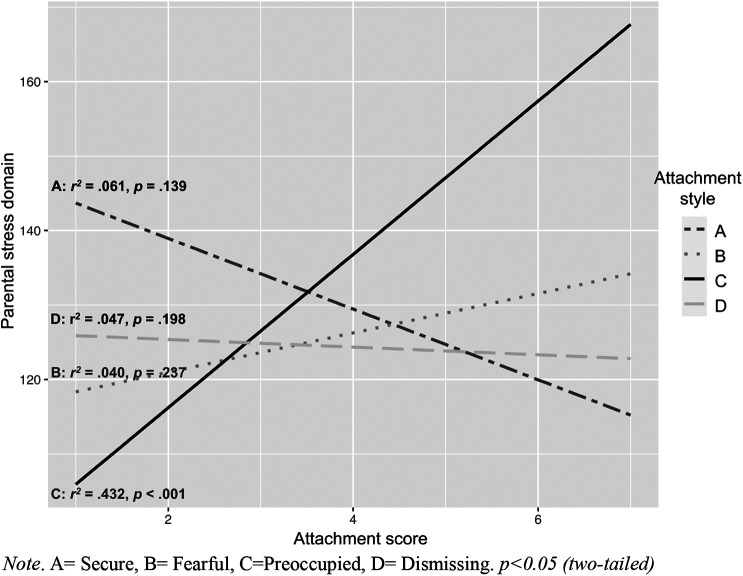
Correlates and regression coefficients of parental attachment styles and parent-related stress. A, Secure; B, Fearful; C, Preoccupied; D, Dismissing. *p* < 0.05 (two-tailed).

**Figure 3:**
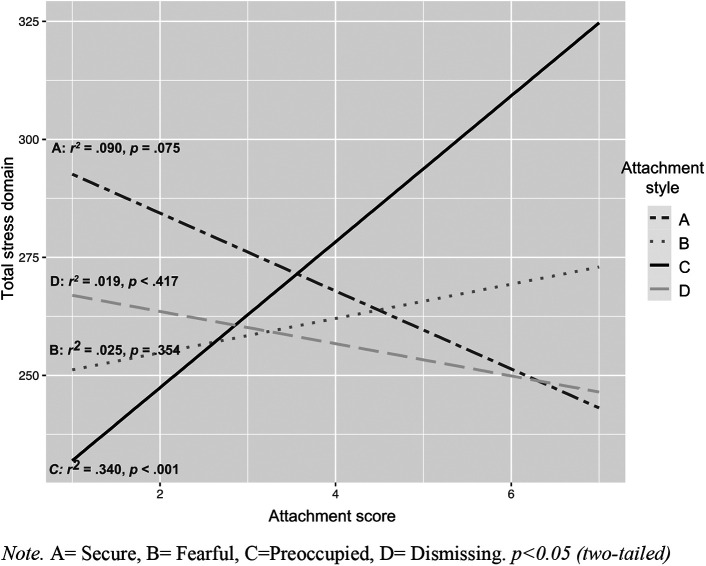
Correlates and regression coefficients of parental attachment styles and total stress load. A, Secure; B, Fearful; C, Preoccupied; D, Dismissing. *p* < 0.05 (two-tailed).

In the regression analyses, conducted separately for each attachment style and stress domain, the variation explained by attachment style C (preoccupied) was 12% (*r*^2^ = 0.12) for child-related stress, 43% (*r*^2^ = 0.43) for parental stress, and 34% (*r*^2^ = 0.34) for the total stress score (higher stress levels correlate with higher associations to style C). Attachment styles A (secure), B (fearful), or D (dismissing) did not significantly affect variation in the child and parent domains, or total stress loads, in the DMDD group (see Table 5). However, types A and D and parenting stress showed a negative tendency in which a higher association with type A and D showed lower parenting stress levels (Figures 1–3).

**Table 5:** Summary of linear regression models for attachment style predicting parenting stress levels in the DMDD group*.*

	*B*	95% CI	*Β*	*t*(1,34)	*p*	*r* ^2^_adj_
*n* = 37		Child stress domain				
Type A (secure)	−3.51	−8.22 to 1.19	−0.248	−1.51	0.139	0.03
Type B (fearful)	0.534	−3.40 to 4.47	0.046	0.275	0.785	−0.02
Type C (preoccupied)	4.76	0.397 to 9.13	0.351	2.21	0.033*	0.10
Type D (dismissing)	−2.72	−6.93 to 1.49	−0.217	−1.31	0.198	0.02
		Parental stress domain				
Type A (secure)	−4.74	−10.1 to 0.618	−0.291	−1.76	0.081	0.05
Type B (fearful)	2.64	−1.81 to 7.09	0.199	1.20	0.237	0.01
Type C (preoccupied)	10.29	6.24 to 14.34	0.657	5.15	<0.001**	0.41
Type D (dismissing)	−0.515	−5.48 to 4.46	−0.035	−0.210	0.835	−0.02
		Total stress				
Type A (secure)	−8.525	−17.37 to 0.868	−0.301	−1.83	0.075	0.06
Type B (fearful)	3.629	−4.21 to 11.47	0.159	0.940	0.354	−0.00
Type C (preoccupied)	15.45	7.95 to 22.95	0.583	4.18	<0.001**	0.32
Type D (dismissing)	−3.413	−11.86 to 5.03	−0.139	−0.821	0.417	−0.00

CI, confidence interval for B.

* *p* < 0.05; **p < 0.001.

## Discussion

The present study's main findings showed that parents of children with DMDD (1) experience higher levels of stress related to demands from their child, (2) describe themselves as significantly more insecure in their adult attachment style, and (3) have a significantly higher association with the insecure type D (dismissing) attachment style than parents of children with other diagnoses. There was no difference between the secure and insecure styles and parental stress derived from the child's demands in the DMDD group (4), but (5) the preoccupied attachment style (C) significantly correlated with and predicted levels of child-related stress, parent-related stress, and total parenting stress to a moderate-to-strong degree. Thus, the results further indicated that perceived parental demands from the child is the main predictor of parental stress in the DMDD group, as opposed to parental attachment styles, but with attachment style C (preoccupied) being a further contributing factor.

Parents of children with DMDD report significantly higher stress levels in relation to their child's behavior or demands than parents of children with other mental health diagnoses. This particular type of stress refers to challenges within the child and its relationship to parenting stress levels, i.e., the degree to which it is experienced as “easy” or “difficult” to parent the child. Children with DMDD have externalizing and internalizing symptoms (33) and are likely to show difficulties in most areas captured in this stress domain. In addition, DMDD is associated with higher symptom loads and lower functional levels in multiple areas than most other child mental health diagnoses (3), most likely increasing stress and worries related to parenting the child.

Furthermore, children with DMDD typically show reactive, intense, and impulsive anger outbursts. The large and unpredictable variation in feelings and behavior may cause uncertainty for the parents about what to expect or demand from the child. Uncertainty produces indecisiveness and a poor ability to prepare, which in turn can lead to anxiety and elevated stress (34). Uncertainty causes even higher stress levels than inevitable pain (35). Indeed, parents of children with DMDD experience high levels of stress related to child factors, regardless of the type of parental attachment.

Another important finding from this study was that a larger percentage of parents of children with DMDD described themselves as significantly more insecure in close relationships with others than parents of children with other disorders. An insecure attachment style was associated with significantly higher levels of parenting stress (related to the demands of being a parent itself and the total stress load of parenting). This finding supports other evidence of the relationship between attachment style and parental stress (36, 37). Indeed, as an adult insecure attachment style is linked to difficulties with emotion regulation and maladaptive responses to distress (38), this might partly explain how stressful parenting tasks are perceived by these parents.

It is difficult to be certain of the direction of the relationship between an insecure parent attachment style and DMDD symptoms. However, several studies have shown that correlations between parenting stress and attachment are most likely bidirectional (21), and a considerable amount of research links insecure attachment to more negative parental behavior (39). Following this line of reasoning, one could suspect that a higher number of parents of children with DMDD are struggling with effective parenting skills.

Interestingly, there was a higher association with attachment style D (dismissing) in the parental DMDD group than in parents with other child mental health diagnoses. Style D showed a negative trend in association to parenting stress in all domains, in which a higher degree of association to type D equals less perceived stress. As type D is described as self-sufficient and dismissing in close relationships (40), such as between a parent and child, one can speculate whether this results in a more detached way of experiencing parenting struggles, and therefore less perceived stress.

A higher degree of association with preoccupied attachment style (C) predicted all parental stress domains in the DMDD group, whereas resemblance to the other attachment styles did not. Although the sample size in the DMDD group was small and the findings should be interpreted with caution, this result might indicate that type C explained most of the variability in parental stress in the insecure DMDD group and that parents associated with this style are at a higher risk of experiencing parental stress in general. Adults with a preoccupied style are thought to be associated with a negative self-image, overly dependent and craving intimacy, emotionally highly expressive, and anxious/ambivalent in close relationships (15). Notably, these traits have been associated with a proneness to elevated stress levels in other studies (21). Style C (preoccupied) in Bartholomew's model is equivalent to other adult attachment measures, such as the ambivalent style in the Adult Attachment Interview (AAI) (17), which have shown specific challenges related to parenting skills, e.g., being inconsistent (41), less authoritative (42), and experiencing childcare as aggravating (43). Altogether, these associations might explain why parenting is perceived as so stressful for parents with type C (preoccupied) attachment style. Owing to the small sample, the findings should be regarded as preliminary and further research is needed to substantiate them.

### Strengths and limitations

This study has several strengths, such as exploring a novel field with children who were systematically and comprehensively assessed at the time of referral, resulting in a well-defined group of children with mental health disorders. Moreover, using a dimensional measure of attachment styles is in accordance with research on adult attachment, implying that categorical measures lack sensitivity to variations in each attachment style (44). However, there are some limitations. First, the sample size and statistical power are low in the DMDD group and may have resulted in type 1 and type 2 errors (45), especially when considering correlations between attachment styles and stress levels. Second, the RQ intently measures romantic attachment style, and conclusions about the relationship with one's child should be made with caution. The AAI (46) is described as the gold standard of adult attachment measurement (47) and could as such be considered for future research. However, the AAI has been criticized for its limited focus on childhood experiences when investigating adult attachment (48). Nonetheless, several studies have shown that self-reported adult attachment questionnaires and the AAI overlap, and therefore most likely measure the same phenomena (44). Future studies that include measurements of attachment styles in children with DMDD (Children Attachment Interview [CAI] [49]) combined with parent attachment measures could increase understanding of the potential impact of attachment in this patient group.

## Conclusion

Parents of children with DMDD experience high levels of parenting stress related to factors in the child and show a higher association with a dismissing insecure adult attachment style than parents of children without DMDD. Although the study’s small sample size must be taken into account, the preoccupied attachment style explains the variability in parental stress in the DMDD group to a high degree. Parent attachment style and parenting stress may negatively influence caregiving practices. Therefore, these findings should be considered when offering help to children with DMDD by including parents more in the treatment programs. There is preliminary evidence that supports parental intervention programs for children with DMDD, such as Dialectical Behavioral Therapy for Children (DBT-C) (50, 51). DBT-C focuses on parents’ emotional regulation, stress reduction, and child–parent interactions (50). The present study’s results may support why such interventions have an effect.

## Data Availability

The datasets presented in this article are not readily available because according to Norwegian legislation, the Norwegian Data Protection Authority, and the Committee of Ethics, we are not allowed to share original clinical study data publicly. Requests to access the datasets should be directed to mmc@lds.no.
