# Supplementary material for: Disruptive mood dysregulation disorder, parental stress, and attachment styles
Source: Front Child Adolesc Psychiatry. 2024 Jul 23;3:1430850. doi: 10.3389/frcha.2024.1430850 (PMC11751584; doi:10.3389/frcha.2024.1430850)
Supplement: Supplementary file 1 [file Table1.docx]

**Supplementary**

**Appendix 1**

Relationship Questionnaire (RQ)

Scale:

Following are four general relationship styles that people often report. Place a checkmark next to the letter corresponding to the style that best describes you or is closest to the way you are.


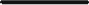
A. It is easy for me to become emotionally close to others. I am comfortable depending on them and having them depend on me. I don't worry about being alone or having others not accept me.


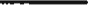
 B. I am uncomfortable getting close to others. I want emotionally close relationships, but I find it difficult to trust others completely, or to depend on them. I worry that I will be hurt if I allow myself to become too close to others.


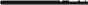
 C. I want to be completely emotionally intimate with others, but I often find that others are reluctant to get as close as I would like. I am uncomfortable being without close relationships, but I sometimes worry that others don't value me as much as I value them.


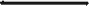
 D. I am comfortable without close emotional relationships. It is very important to me to feel independent and self-sufficient, and I prefer not to depend on others or have others depend on me.

Now please rate each of the relationship styles above to indicate how well or poorly each description corresponds to your general relationship style.

# Style A

1 2 3 4 5 6 7

Disagree Neutral/

Strongly Mixed Strongly

# style B

1 2 3 4 5 6 7

Disagree Neutral/ Agree

Strongly Mixed Strongly

# style C

1 2 3 4 5 6 7

Disagree Neutral/ Agree

Strongly Mixed Strongly

# style D

1 2 3 4 5 6 7

Disagree Neutral/

Strongly Mixed Strongly

**Table 1**

*Perceived Stress Related to the Child, Parent and Total Stress Load as Reported by Parents and Parent Attachment Style for Children with DMDD Compared to Other Major Disorders*

|  | *M* (SD) | *M* (SD) | Test statistic  *t*(df), *χ*^2^ | *P*-value  (two-tailed) | |
| --- | --- | --- | --- | --- | --- |
|  | **DMDD vs ADHD^a^** | | |  | |
| **Parental stress** | (*n*=17) | (*n*=48) |  |  | |
| Child domain | 129.7(21.9) | 128.0 (21.0) | *t*(63)=.290 | | .773 |
| Parent domain | 119.8(25.7) | 119.2(23.6) | *t*(63)=.090 | | .929 |
| Total stress load | 249.5(43.8) | 247.2(35.3) | *t*(63)=.224 | | .824 |
| **Parent Attachment Style** | (*n*=15) | (*n*=42) |  | |  |
| **Secure** (*n*,%) | 9(60) | 31(73.8) | *χ*^2^_(1,57)_=1.01 | | .341 |
| I**nsecure** (*n*,%) | 6(40) | 11(26.2) |  | |  |
| **Attachment types** | (*n*=15) | (*n*=42) |  | |  |
| Type A (Secure) | 5.4(1.8) | 5.1(1.5) | *t*(55)=.477 | | .635 |
| Type B (Fearful) | 3.6(2.0) | 2.9(1.8) | *t*(55)=1.22 | | .227 |
| Type C (Preoccupied) | 2.2(1.6) | 2.6(1.6) | *t*(55)=.872 | | .387 |
| Type D (Dismissing) | 3.3(1.8) | 2.4(1.4) | *t*(55)=1.92 | | .059 |
|  | **DMDD vs anxiety disorders^a^** | | |  | |
| **Parental stress** | (*n*=32) | (*n*=43) |  |  | |
| Child domain | 131.4(21.9) | 123.0 (21.0) | *t*(73)=1.50 | | .130 |
| Parent domain | 123.1(25.7) | 115.9(23.6) | *t*(73)=1.22 | | .291 |
| Total stress load | 254.5(43.8) | 240.6(35.3) | *t*(73)=1.38 | | .167 |
| **Parent Attachment Style** | (*n*=28) | (*n*=39) |  | |  |
| **Secure** (*n*,%) | 13(46) | 25(64) | *χ*^2^_(1, 67)_=2.07 | | .150 |
| I**nsecure** (*n*,%) | 15(54) | 14(36) |  | |  |
| **Attachment types** | (*n*=28) | (*n*=39) |  | |  |
| Type A (Secure) | 5.0(1.7) | 4.8(2.2) | *t*(65)=.418 | | .677 |
| Type B (Fearful) | 3.5(2.0) | 3.3(2.0) | *t*(65)=.496 | | .621 |
| Type C (Preoccupied) | 2.3(1.5) | 2.9(1.7) | *t*(65)=1.34 | | .183 |
| Type D (Dismissing) | 3.4(1.8) | 2.2(1.3) | *t*(65)=3.06 | | .**003*** |
|  | **DMDD vs depressive disorders^a^** | | |  | |
| **Parental stress** | (*n*=40) | (*n*=12) |  |  | |
| Child domain | 135.2(23.3) | 123.3 (29.0) | t(50)=1.46 | | .213 |
| Parent domain | 124.1(27.8) | 105.5(24.0) | t(50)=2.08 | | .291 |
| Total stress load | 259.2(46.8) | 228.7(51.1) | t(50)=1.93 | | .059 |
| **Parent Attachment Style** | (*n*=33) | (*n*=10) |  | |  |
| **Secure** (*n*,%) | 17(51.5) | 7(70) | *χ*^2^_(1, 43)_=1.06 | | .470 |
| I**nsecure** (*n*,%) | 16(48.5) | 3(30) |  | |  |
| **Attachment types** | (*n*=34) | (*n*=10) |  | |  |
| Type A (Secure) | 5.0(1.6) | 5.3(1.4) | *t*(42)=.510 | | .581 |
| Type B (Fearful) | 3.3(1.9) | 2.7(1.9) | *t*(42)=.960 | | .343 |
| Type C (Preoccupied) | 2.7(1.7) | 2.5(2.0) | *t*(42)=.362 | | .719 |
| Type D (Dismissing) | 3.0(1.7) | 2.2(1.3) | *t*(42)=1.38 | | .068 |
|  | **DMDD vs ODD^a^** | | |  | |
| **Parental stress** | (*n*=44) | (*n*=35) |  |  | |
| Child domain | 135.5(22.2) | 138.7 (16.4) | *t*(77)=.708 | | .481 |
| Parent domain | 124.3(27.4) | 120.1(24.6) | *t*(77)=.720 | | .473 |
| Total stress load | 254.3(59.8) | 258.8(35.3) | *t*(77)=.390 | | .697 |
| **Parent Attachment Style** | (*n*=38) | (*n*=29) |  | |  |
| **Secure** (*n*,%) | 19(50) | 20(69) | *χ*^2^_(1, 67)_=2.43 | | .140 |
| I**nsecure** (*n*,%) | 19(50) | 9(31) |  | |  |
| **Attachment types** | (*n*=39) | (*n*=29) |  | |  |
| Type A (Secure) | 5.0(1.6) | 4.9(1.7) | *t*(66)=.296 | | .769 |
| Type B (Fearful) | 3.5(2.0) | 3.2(2.0) | *t*(66)=.526 | | .601 |
| Type C (Preoccupied) | 2.7(1.6) | 3.2(1.8) | *t*(66)=1.03 | | .304 |
| Type D (Dismissing) | 3.0(1.7) | 2.0(1.3) | *t*(66)=2.71 | | .**006*** |

Note. ^a^ **=** comparing diagnoses excludes the other diagnose. DMDD= disruptive mood dysregulation disorder. ADHD= attention deficit hyperactivity disorder, all types. ODD= oppositional disruptive disorder.* = *p* <.005, two-sided.
